# Supplementary material for: Heterologous Production of Isopropanol Using Metabolically Engineered Acetobacterium woodii Strains
Source: Bioengineering (Basel). 2023 Nov 30;10(12):1381. doi: 10.3390/bioengineering10121381 (PMC10741115; doi:10.3390/bioengineering10121381)
Supplement: Supplementary file 1 [file bioengineering-10-01381-s001.zip › bioengineering-2733126-supplementary.pdf]

bioengineering

Supplementary Materials

Heterologous Production of Isopropanol Using Metabolically Engineered *Acetobacterium woodii* Strains

Franziska Höfele [1,*], Teresa Schoch [2], Catarina Oberlies [2] and Peter Dürre [2]

[1] Institute of Molecular Biology and Biotechnology of Prokaryotes, Ulm University, 89081 Ulm, Germany
[2] Institute of Microbiology and Biotechnology, Ulm University, 89081 Ulm, Germany; catarina.oberlies@gmail.com (C.O.); peter.duerre@uni-ulm.de (P.D.)
* Correspondence: franziska.hoefele@uni-ulm.de

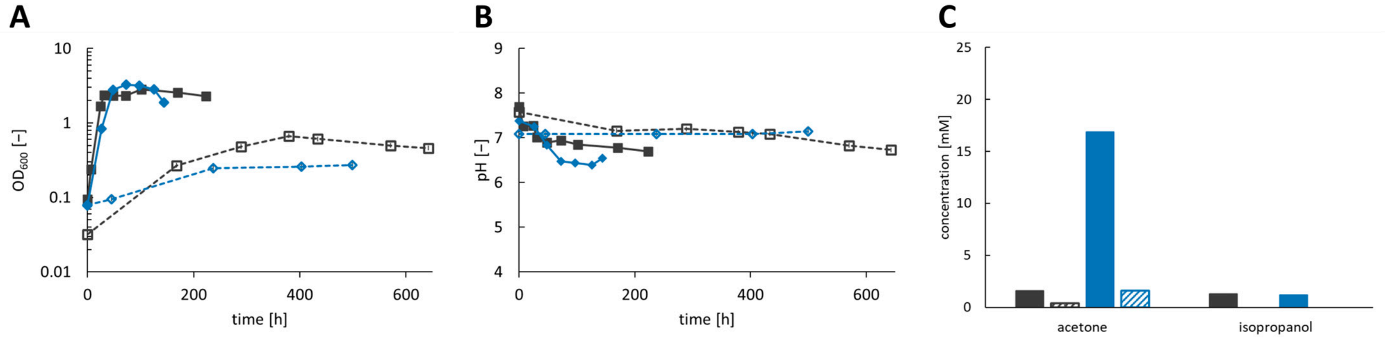

Figure S1. Production of acetone and isopropanol using recombinant *A. woodii* strains, harboring the acetone production pathway, cultivated with 60 mM fructose or $CO_2 + H_2$ as substrate. During growth (**A**) OD_{600}, (**B**) changes in pH, and (**C**) maximum product concentrations of acetone and isopropanol were monitored. The cultivation of *A. woodii* [pJIR750_ac2t2] using fructose or $CO_2 + H_2$ is depicted as grey lines with filled rectangles and dashed grey lines with empty rectangles, respectively. The growth of the recombinant strain *A. woodii* [pJIR750_ac3t3] with fructose or $CO_2 + H_2$ is depicted as blue line with filled rhombus and dashed blue line with empty rhombus, respectively. Growth of the strains was not performed in parallel and in the same charge of medium.

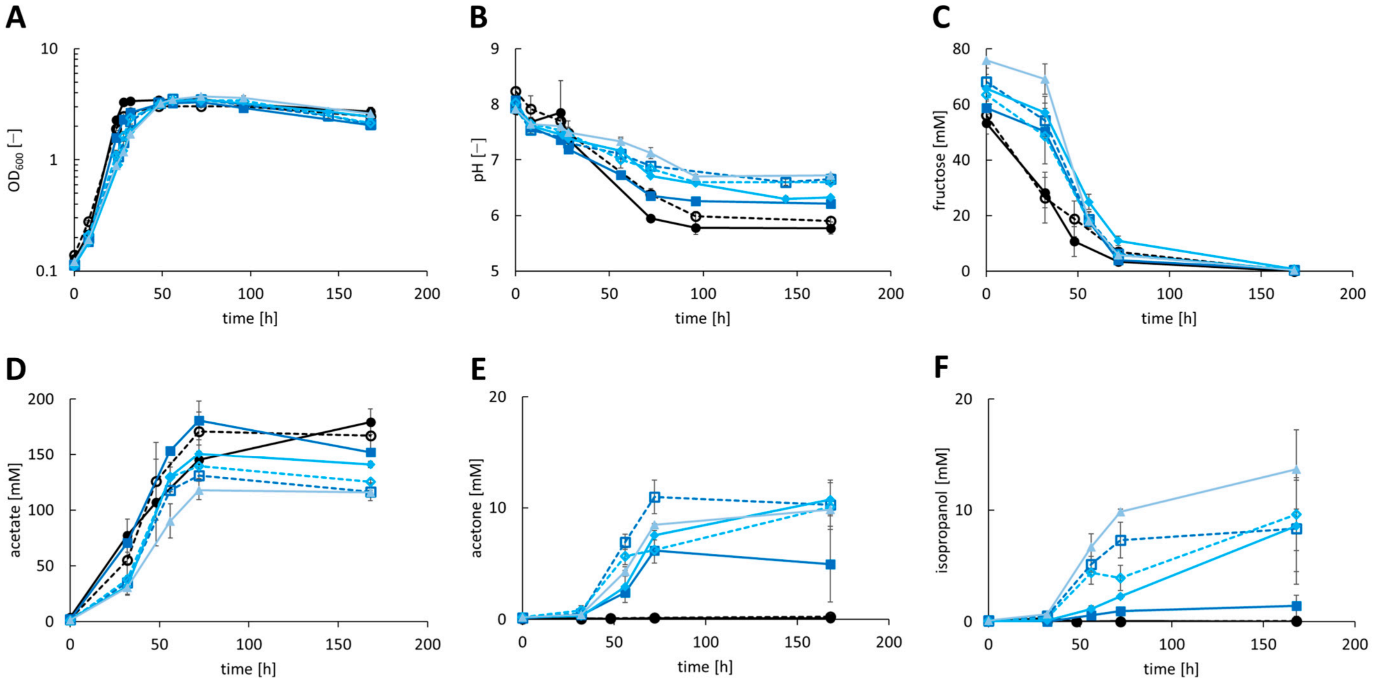

Figure S2. Heterotrophic production of isopropanol using recombinant *A. woodii* strains, harboring the secondary alcohol dehydrogenase (*sadH*) gene of *C. beijerinckii* DSM 6423, cultivated with 60 mM fructose as substrate. Growth was performed in biological triplicates with *A. woodii* wild-type and *A. woodii* [pJIR750] as control strains (depicted as black line with filled circles and dashed black line with empty circles, respectively). During growth (**A**) OD_{600}, (**B**) changes in pH, (**C**) fructose consumption, and production of (**D**) acetate, (**E**) acetone, and (**F**) isopropanol were monitored. The recombinant strains *A. woodii* [pJIR750_ac1t1s1] and *A. woodii* [pJIR750_ac1t2s1] are depicted as dark blue lines with filled rectangles and dashed dark blue lines with empty rectangles, respectively. *A. woodii* [pJIR750_ac2t1s1] and *A. woodii* [pJIR750_ac2t2s1] are shown as light blue lines with filled rhombus and dashed light blue lines with empty rhombus, respectively. The pastel blue lines with filled triangle depict *A. woodii* [pJIR750_ac3t3s1].

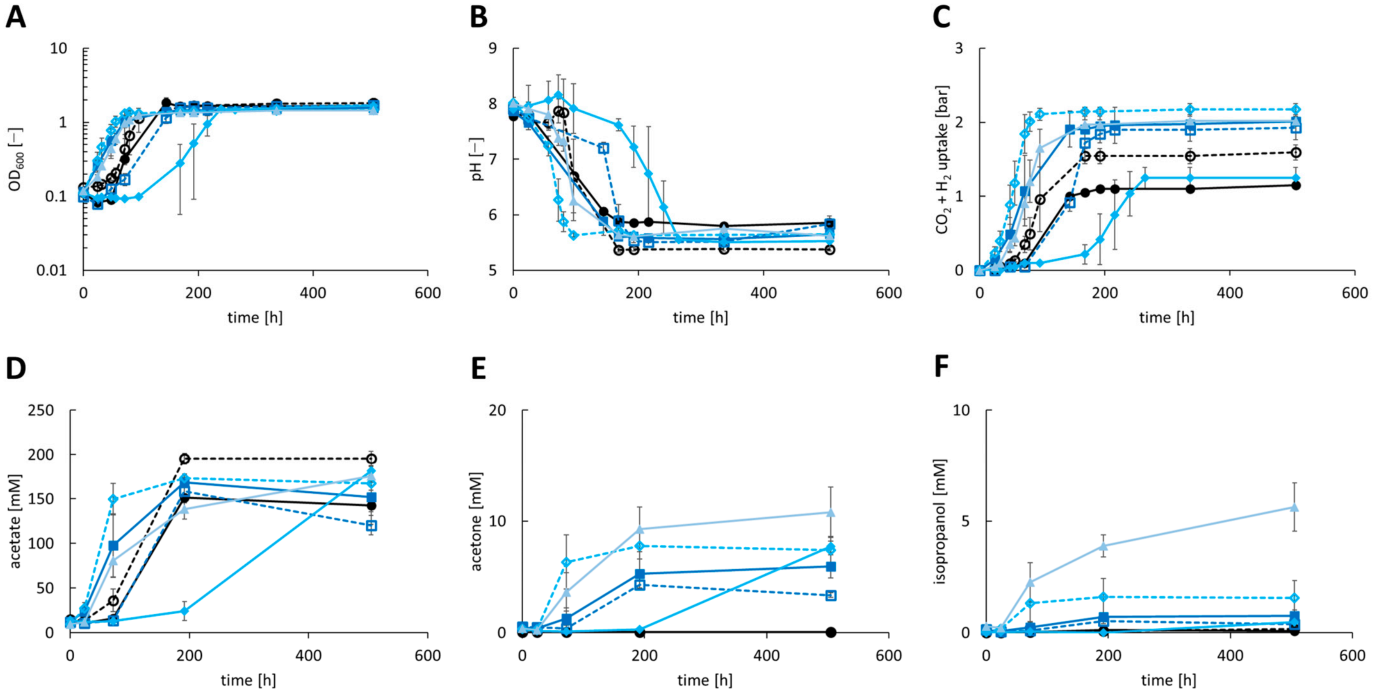

Figure S3. Autotrophic production of isopropanol using recombinant *A. woodii* strains, harboring the *sadH* gene of *C. beijerinckii* DSM 6423, cultivated with CO_2 + H_2 as substrate. Growth was performed in biological triplicates with *A. woodii* wild-type and *A. woodii* [pJIR750] as control strains (depicted as black line with filled circles and dashed black line with empty circles, respectively). During growth (**A**) OD_{600}, (**B**) changes in pH, (**C**) fructose consumption, and production of (**D**) acetate, (**E**) acetone, and (**F**) isopropanol were monitored. The recombinant strains *A. woodii* [pJIR750_ac1t1s1] and *A. woodii* [pJIR750_ac1t2s1] are depicted as dark blue lines with filled rectangles and dashed dark blue lines with empty rectangles, respectively. *A. woodii* [pJIR750_ac2t1s1] and *A. woodii* [pJIR750_ac2t2s1] are shown as light blue lines with filled rhombus and dashed light blue lines with empty rhombus, respectively. The pastel blue lines with filled triangle depict *A. woodii* [pJIR750_ac3t3s1].

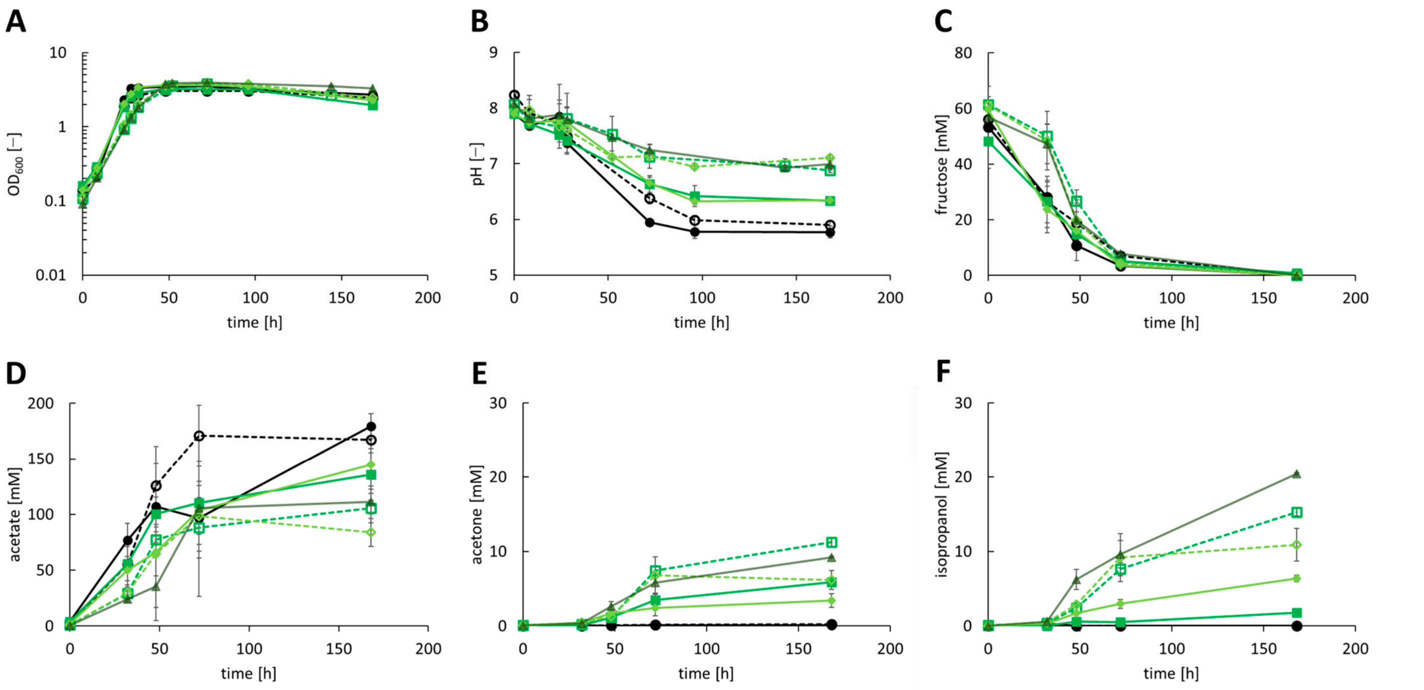

Figure S4. Heterotrophic production of isopropanol using recombinant *A. woodii* strains, harboring the *sadH* gene of *C. beijerinckii* DSM 15410, cultivated with 60 mM fructose as substrate. Growth was performed in biological triplicates with *A. woodii* wild-type and *A. woodii* [pJIR750] as control strains (depicted as black line with filled circles and dashed black line with empty circles, respectively). During growth (**A**) OD_{600}, (**B**) changes in pH, (**C**) fructose consumption, and production of (**D**) acetate, (**E**) acetone, and (**F**) isopropanol were monitored. The recombinant strains *A. woodii* [pJIR750_ac1t1s2] and *A. woodii* [pJIR750_ac1t2s2] are depicted as green lines with filled rectangles and dashed green lines with empty rectangles, respectively. *A. woodii* [pJIR750_ac2t1s2] and *A. woodii* [pJIR750_ac2t2s2] are shown as light green lines with filled rhombus and dashed light green lines with empty rhombus, respectively. The brown-green lines with filled triangle depict *A. woodii* [pJIR750_ac3t3s2].

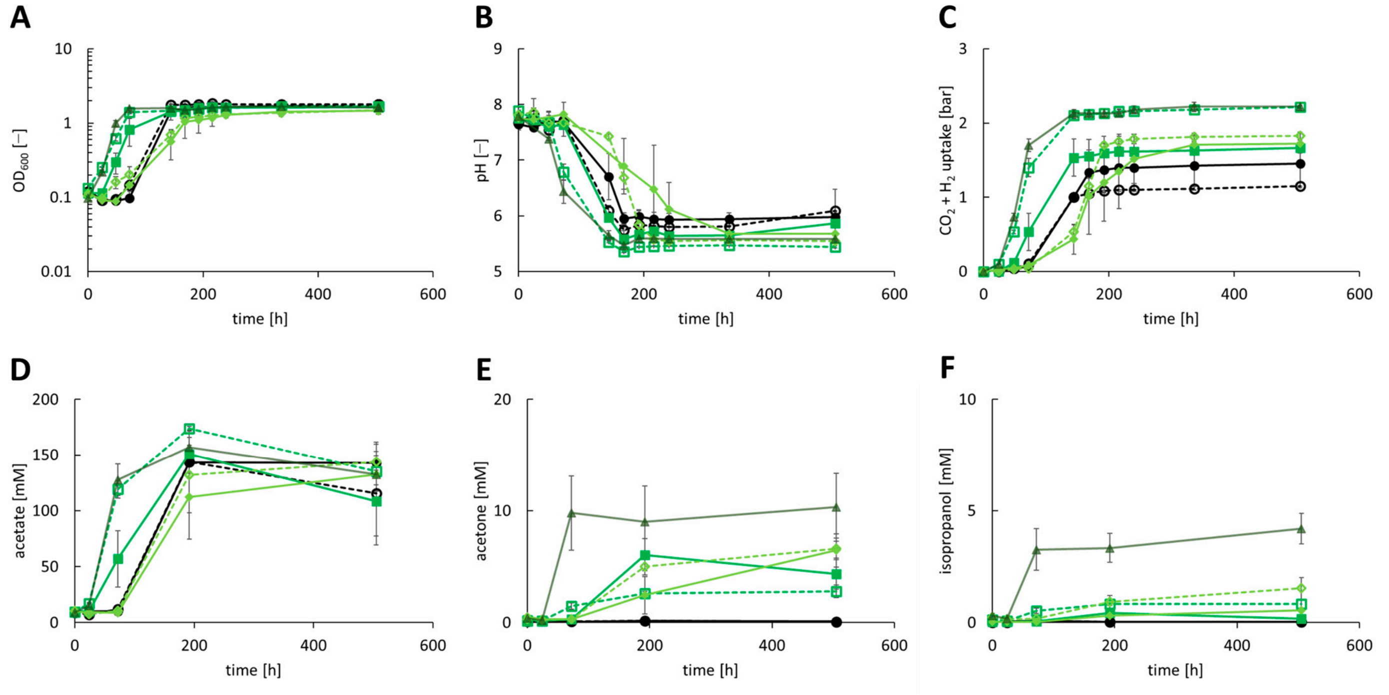

Figure S5. Autotrophic production of isopropanol using recombinant *A. woodii* strains, harboring the *sadH* gene of *C. beijerinckii* DSM 15410, cultivated with CO_2 + H_2 as substrate. Growth was performed in biological triplicates with *A. woodii* wild-type and *A. woodii* [pJIR750] as control strains (depicted as black line with filled circles and dashed black line with empty circles, respectively). During growth (**A**) OD_{600}, (**B**) changes in pH, (**C**) fructose consumption, and production of (**D**) acetate, (**E**) acetone, and (**F**) isopropanol were monitored. The recombinant strains *A. woodii* [pJIR750_ac1t1s2] and *A. woodii* [pJIR750_ac1t2s2] are depicted as green lines with filled rectangles and dashed green lines with empty rectangles, respectively. *A. woodii* [pJIR750_ac2t1s2] and *A. woodii* [pJIR750_ac2t2s2] are shown as light green lines with filled rhombus and dashed light green lines with empty rhombus, respectively. The brown-green lines with filled triangle depict *A. woodii* [pJIR750_ac3t3s2].

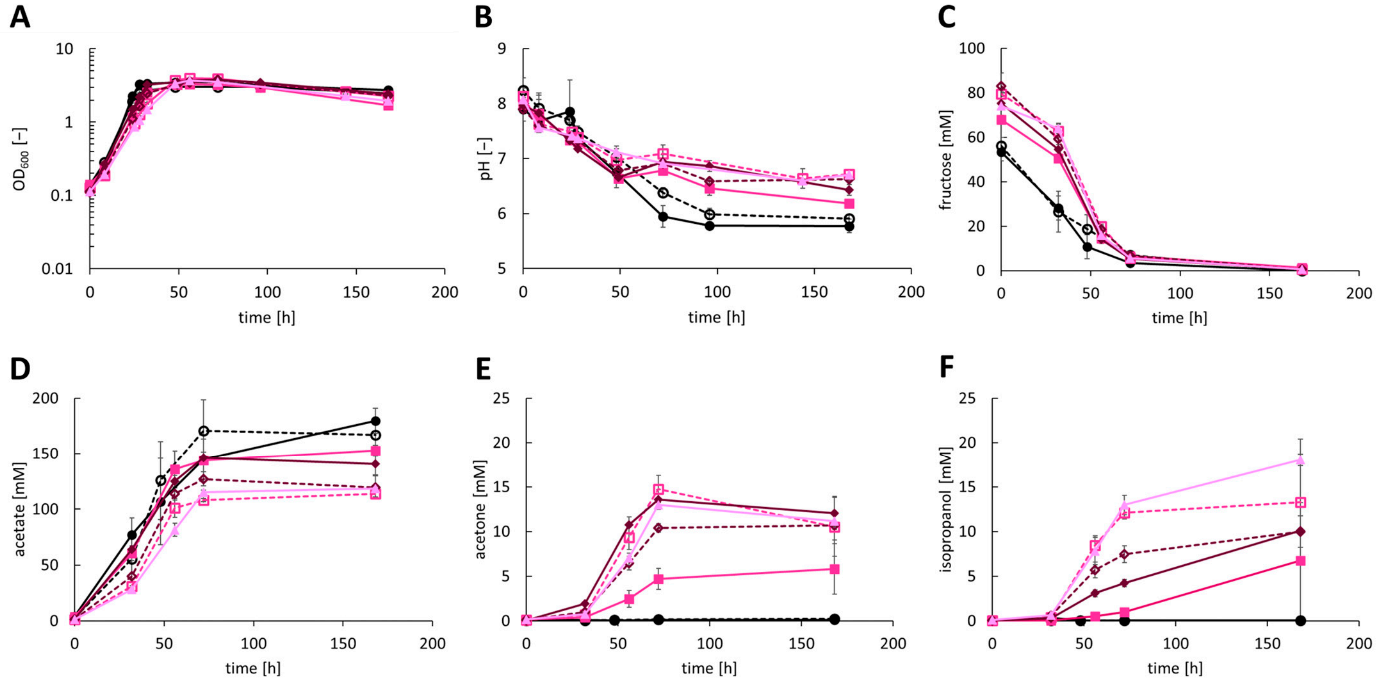

Figure S6. Heterotrophic production of isopropanol using recombinant *A. woodii* strains, harboring the *sadH* gene of *C. ljungdahlii*, cultivated with 60 mM fructose as substrate. Growth was performed in biological triplicates with *A. woodii* wild-type and *A. woodii* [pJIR750] as control strains (depicted as black line with filled circles and dashed black line with empty circles, respectively). During growth (**A**) OD_{600}, (**B**) changes in pH, (**C**) fructose consumption, and production of (**D**) acetate, (**E**) acetone, and (**F**) isopropanol were monitored. The recombinant strains *A. woodii* [pJIR750_ac1t1s3] and *A. woodii* [pJIR750_ac1t2s3] are depicted as pink lines with filled rectangles and dashed pink lines with empty rectangles, respectively. *A. woodii* [pJIR750_ac2t1s3] and *A. woodii* [pJIR750_ac2t2s3] are shown as purple lines with filled rhombus and dashed purple lines with empty rhombus, respectively. The rosa lines with filled triangle depict *A. woodii* [pJIR750_ac3t3s3].

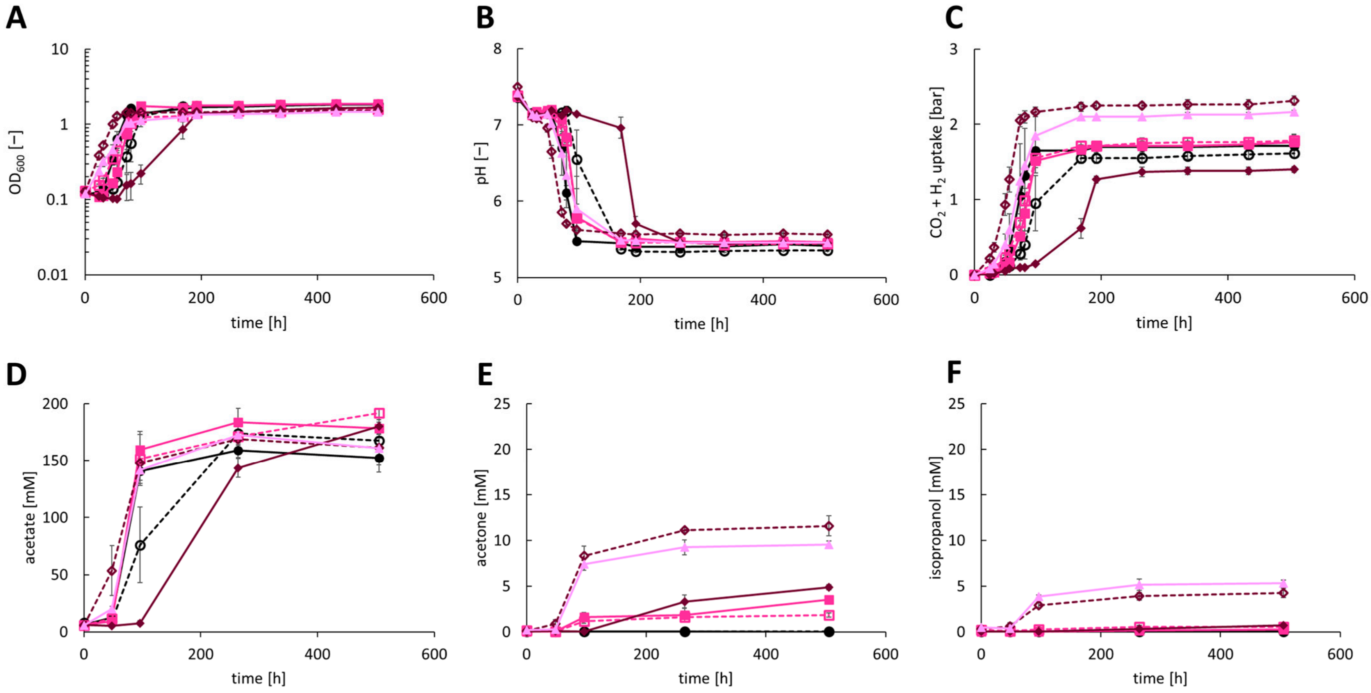

Figure S7. Autotrophic production of isopropanol using recombinant *A. woodii* strains, harboring the *sadH* gene of *C. ljungdahlii*, cultivated with CO_2 + H_2 as substrate. Growth was performed in biological triplicates with *A. woodii* wild-type and *A. woodii* [pJIR750] as control strains (depicted as black line with filled circles and dashed black line with empty circles, respectively). During growth (**A**) OD_{600}, (**B**) changes in pH, (**C**) fructose consumption, and production of (**D**) acetate, (**E**) acetone, and (**F**) isopropanol were monitored. The recombinant strains *A. woodii* [pJIR750_ac1t1s3] and *A. woodii* [pJIR750_ac1t2s3] are depicted as pink lines with filled rectangles and dashed pink lines with empty rectangles, respectively. *A. woodii* [pJIR750_ac2t1s3] and *A. woodii* [pJIR750_ac2t2s3] are shown as purple lines with filled rhombus and dashed purple lines with empty rhombus, respectively. The rosa lines with filled triangle depict *A. woodii* [pJIR750_ac3t3s3].

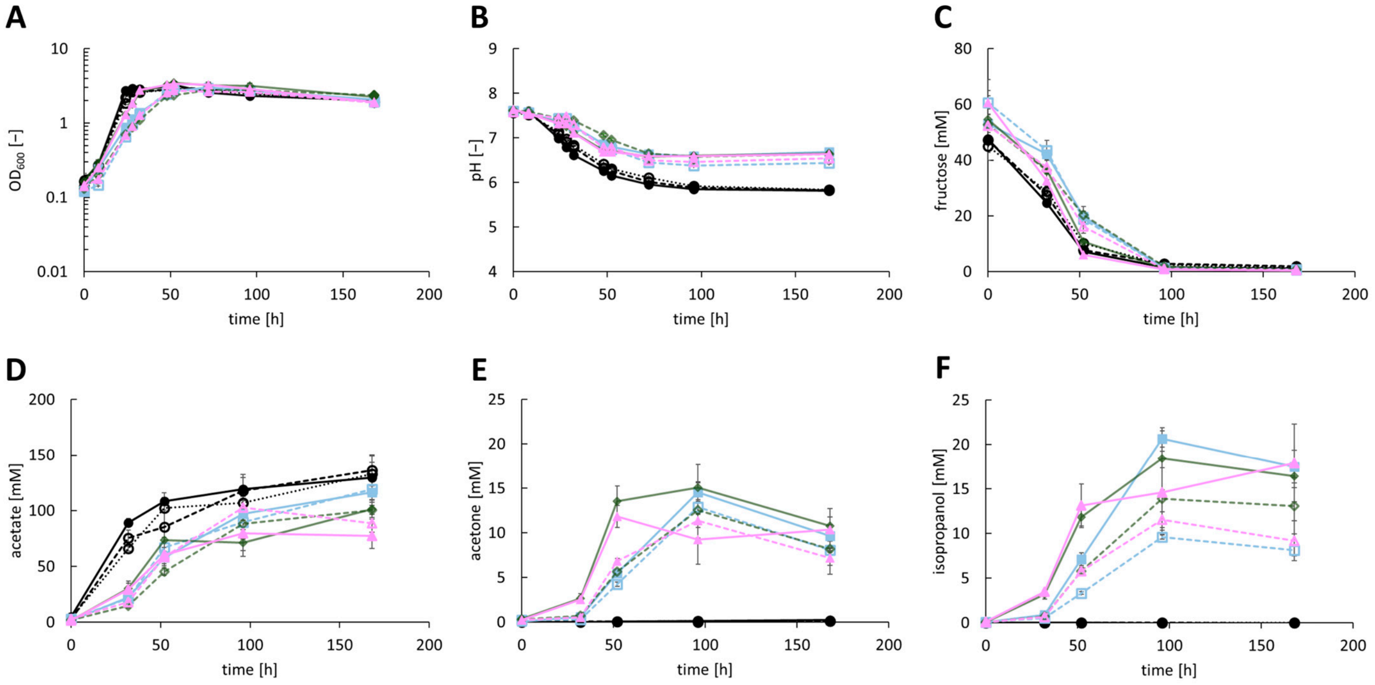

Figure S8. Heterotrophic production of isopropanol using recombinant *A. woodii* strains, harboring the *hydG* gene of *C. beijerinckii* DSM 6423, cultivated with 60 mM fructose as substrate. Growth was performed in biological triplicates with *A. woodii* wild-type and *A. woodii* [pJIR750] as control strains (depicted as black line with filled circles and dashed black line with empty circles, respectively). During growth (**A**) OD_{600}, (**B**) changes in pH, (**C**) fructose consumption, and production of (**D**) acetate, (**E**) acetone, and (**F**) isopropanol were monitored. The recombinant strains *A. woodii* [pJIR750_ac3t3s1] and *A. woodii* [pJIR750_ac3t3s1] [pMTL83251_PthlA_h1] are depicted as pastel blue lines with filled rectangles and dashed pastel blue lines with empty rectangles, respectively. *A. woodii* [pJIR750_ac3t3s2] and *A. woodii* [pJIR750_ac3t3s2] [pMTL83251_PthlA_h1] are shown as brown-green lines with filled rhombus and dashed brown-green lines with empty rhombus, respectively. The rosa lines with filled triangle and dashed rosa lines with empty rectangles depict *A. woodii* [pJIR750_ac3t3s3] and *A. woodii* [pJIR750_ac3t3s3] [pMTL83251_PthlA_h1], respectively.

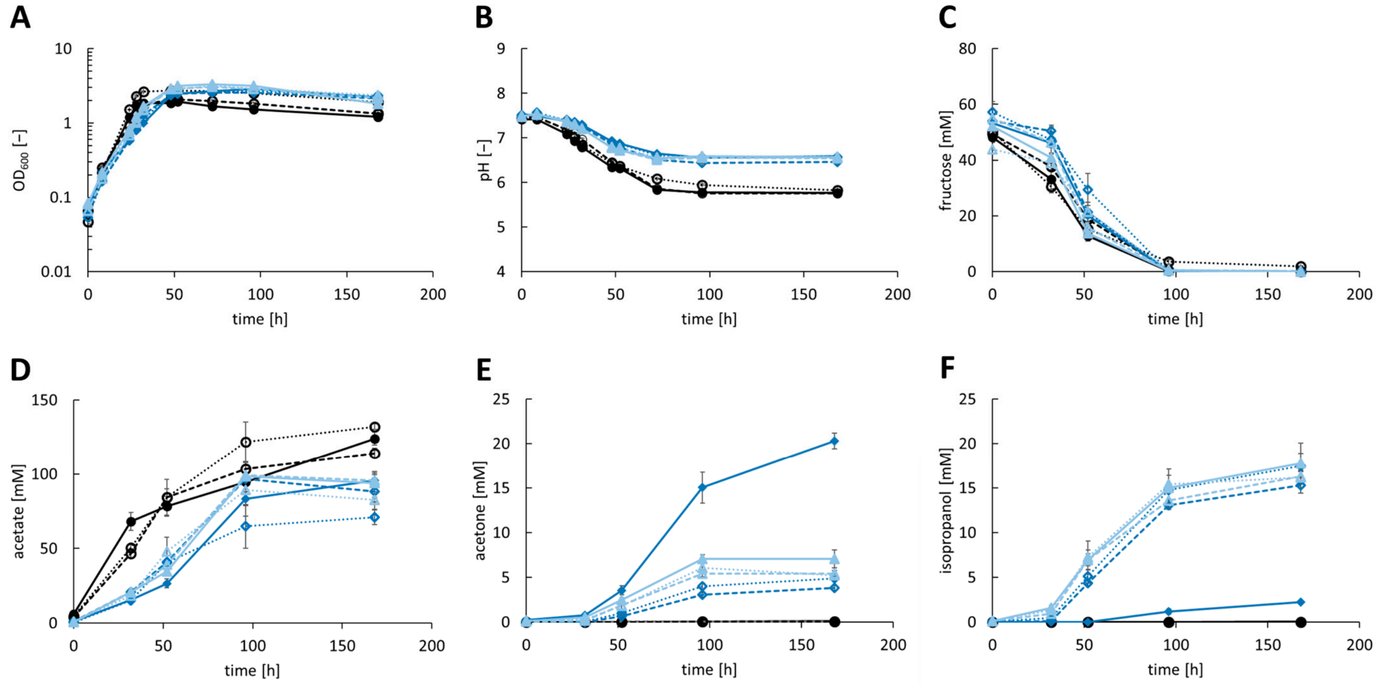

Figure S9. Heterotrophic production of isopropanol using recombinant *A. woodii* strains, harboring the *sadH-hydG* gene of *C. beijerinckii* DSM 6423, cultivated with 60 mM fructose as substrate. Growth was performed in biological triplicates with *A. woodii* wild-type and *A. woodii* [pJIR750] as control strains (depicted as black line with filled circles and dashed black line with empty circles, respectively). During growth (**A**) OD_{600}, (**B**) changes in pH, (**C**) fructose consumption, and production of (**D**) acetate, (**E**) acetone, and (**F**) isopropanol were monitored. The recombinant strains *A. woodii* [pJIR750_ac3t3], *A. woodii* [pJIR750_ac3t3] [pMTL83251_PthlA_sh1], and *A. woodii* [pJIR750_ac3t3] [pMTL83251_PthlA_sh1c3] are depicted as dark blue lines with filled rhombus, dashed dark blue lines with empty rhombus, and dotted dark blue lines with empty rhombus, respectively. *A. woodii* [pJIR750_ac3t3s1], *A. woodii* [pJIR750_ac3t3s1] [pMTL83251_PthlA_sh1], and *A. woodii* [pJIR750_ac3t3s1] [pMTL83251_PthlA_sh1c3] are shown as pastel blue lines with filled triangles, dashed pastel blue lines with empty triangles, and dotted pastel blue lines with empty triangles, respectively.

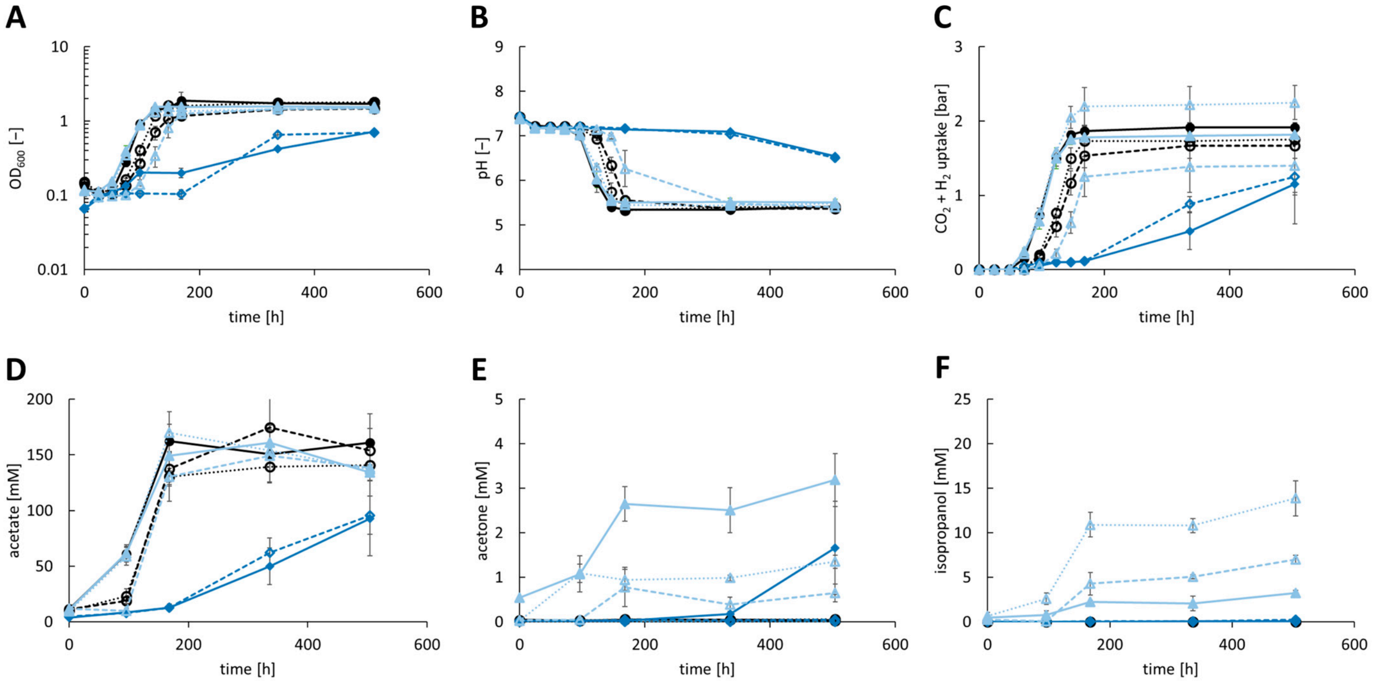

Figure S10. Autotrophic production of isopropanol using recombinant *A. woodii* strains, harboring the *sadH-hydG* gene of *C. beijerinckii* DSM 6423, cultivated with CO_2 + H_2 as substrate. Growth was performed in biological triplicates with *A. woodii* wild-type and *A. woodii* [pJIR750] as control strains (depicted as black line with filled circles and dashed black line with empty circles, respectively). During growth (**A**) OD_{600}, (**B**) changes in pH, (**C**) fructose consumption, and production of (**D**) acetate, (**E**) acetone, and (**F**) isopropanol were monitored. The recombinant strains *A. woodii* [pJIR750_ac3t3], *A. woodii* [pJIR750_ac3t3] [pMTL83251_PthlA_sh1], and *A. woodii* [pJIR750_ac3t3] [pMTL83251_PthlA_sh1c3] are depicted as dark blue lines with filled rhombus, dashed dark blue lines with empty rhombus, and dotted dark blue lines with empty rhombus, respectively. *A. woodii* [pJIR750_ac3t3s1], *A. woodii* [pJIR750_ac3t3s1] [pMTL83251_PthlA_sh1], and *A. woodii* [pJIR750_ac3t3s1] [pMTL83251_PthlA_sh1c3] are shown as pastel blue lines with filled triangles, dashed pastel blue lines with empty triangles, and dotted pastel blue lines with empty triangles, respectively.
